# Factors influencing the inappropriate dosing of rivaroxaban and edoxaban in Chinese hospitalized patients with atrial fibrillation

**DOI:** 10.3389/fcvm.2025.1694976

**Published:** 2026-01-13

**Authors:** Ying Bai, Jianqi Wang, Guangyao Li, Zhen Zhou

**Affiliations:** 1 Department of Pharmacy, Beijing Tongren Hospital, Capital Medical University, Beijing, China; 2 Department of Cardiovascular Center, Beijing Tongren Hospital, Capital Medical University, Beijing, China; 3 School of Biomedical Engineering, Capital Medical University, Beijing, China

**Keywords:** atrial fibrillation, direct oral anticoagulant (DOAC), inappropriate dosing, medication review, risk factors

## Abstract

**Objectives:**

Inappropriate dosing of direct oral anticoagulants (DOACs) may increase the risk of thromboembolism or bleeding in patients with atrial fibrillation (AF). The inappropriate use of these medications presents a significant clinical challenge. Our study aimed to analyze the current utilization of rivaroxaban and edoxaban among Chinese patients with AF, as well as the factors influencing the use of nonstandard doses.

**Methods:**

This study evaluated patients diagnosed with AF between January 2017 and December 2023. Descriptive analyses were performed to summarize the characteristics of the study population. Inappropriate dosing was identified based on the guidelines. Multivariate analysis was performed to identify factors associated with inappropriate dosing in these patients.

**Results:**

A total of 1,066 patients diagnosed with AF, comprising 852 individuals treated with rivaroxaban and 214 individuals treated with edoxaban, were included. Their median age was 69 years, and 58.7% of them were males. Among them, 573 patients (53.8%) received inappropriate dosages. Among the patients prescribed rivaroxaban, 503 (59.0%) were underdosed and eight (0.9%) were overdosed. Among the patients prescribed edoxaban, 49 patients (22.9%) were underdosed and 13 patients (6.1%) were overdosed. Multivariate analysis identified independent factors associated with inappropriate medication dosing, including advanced age [adjusted odds ratio (OR) 1.031, 95% confidence interval (CI) 1.010–1.052], combined use of antiplatelet drugs (adjusted OR 1.649, 95% CI 1.111–2.447), and reduced use of dronedarone (adjusted OR 0.332, 95% CI 0.126–0.877).

**Conclusions:**

The incidence of inappropriate DOAC dosing in Chinese patients with AF was high. Advanced age, the concurrent use of antiplatelet medications, and the nonuse of dronedarone have been identified as independent factors associated with inappropriate dosing.

## 1 Introduction

Atrial fibrillation (AF) is the most prevalent type of sustained cardiac arrhythmia (1). Approximately 30% of individuals with AF are hospitalized at least once per year, with 10% requiring more than two admissions (2–4). Furthermore, AF is associated with a fivefold increase in the risk of ischemic stroke. Guidelines recommended antithrombotic therapy with an oral anticoagulant (OAC) as the primary regimen for thrombotic prevention in patients with AF, effectively decreasing the incidence of cardioembolic events and stroke among high-risk individuals (1, 5, 6).

Warfarin is a classic anticoagulant typically used to prevent thrombotic events in patients with AF and remains one of the most widely prescribed anticoagulants globally. Warfarin has been shown to significantly reduce the risk of stroke in patients with non-valvular AF, with reported efficacy rates reaching up to 64% (7). Despite being a well-established anticoagulant, warfarin also has several limitations that restrict its utilization, such as the slow onset of action, the necessity for regular international normalized ratio monitoring, and a narrow therapeutic range. The optimal dosage of anticoagulant can vary significantly among individuals. In addition to dietary choices, medications, and comorbidities that may impact warfarin metabolism, genetic factors also contribute to individual variability in the required warfarin dosing (8).

The emergence of direct oral anticoagulants (DOACs) has offered new treatment approaches for preventing thromboembolism in patients with AF and has gradually become a prevailing trend in the treatment of AF. These DOACs include direct anti-Xa inhibitors such as apixaban, rivaroxaban, and edoxaban, along with the direct thrombin inhibitor dabigatran, which offers significant advantages over traditional potions like warfarin. A meta-analysis has revealed that compared with warfarin, DOACs are associated with a 19% reduction in the risk of stroke and systemic embolic events, a 10% decrease in all-cause mortality, and a 52% lower risk of hemorrhagic stroke, but a 25% increased risk of gastrointestinal bleeding risk (9). Currently, DOACs are recognized as the first-line treatment for stroke prevention in patients with AF (1, 5, 6). Contrary to warfarin, DOACs provide a more predictable therapeutic effect with a fixed-dose regimen due to their predictable pharmacokinetic and pharmacodynamic profiles. In addition, they do not require routine monitoring and are associated with fewer drug–drug and drug–food interactions (10).

A study conducted in China (2013–2014; 2015–2016) demonstrated a gradual increase in OAC prescription rates for AF patients, increasing from 21.0% to 41.0% over time (11). A systematic review indicated that suboptimal utilization of anticoagulants remains a persisting challenge, despite the availability of DOACs (12). To reduce the risk of bleeding and other adverse effects associated with DOACs, physicians may prescribe low-dose DOACs to patients. However, underdosing of DOAC can be linked to an elevated risk of thromboembolic stroke (13, 14).

To the best of our knowledge, this study is the first to evaluate the factors influencing inappropriate DOAC doses in Chinese hospitalized patients with AF. The study findings are expected to provide valuable insights and recommendations for enhancing the appropriate use of DOAC drugs.

## 2 Patients and methods

### 2.1 Study design

**Figure 1:**
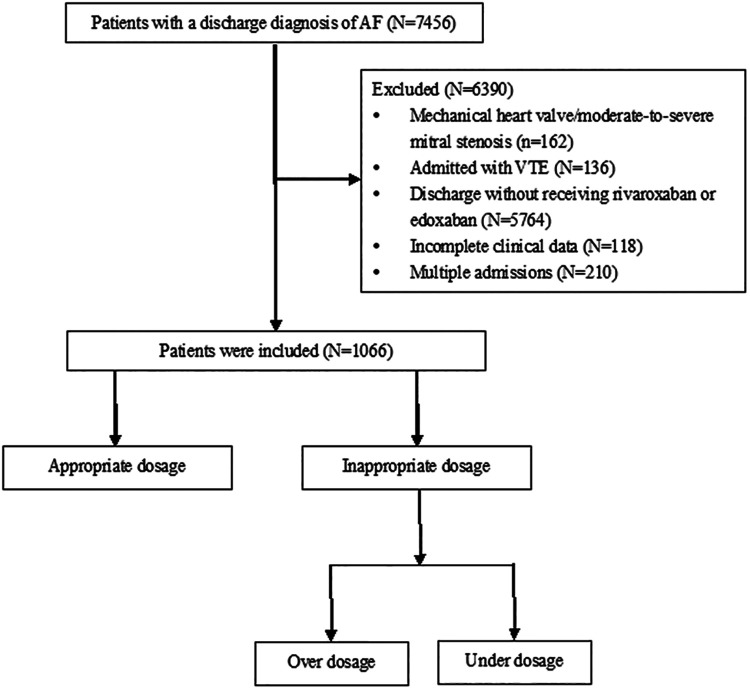
Flow chart of study population.

This retrospective, single-center study was conducted at the Beijing Tongren Hospital, a tertiary hospital with 1,700 beds in China. Ethical approval for this study was obtained from the Ethics Committee of Beijing Tongren Hospital (approval no.: TREC2024-KY152). Given its retrospective nature, the necessity for obtaining written informed consent from patients was waived. This study included individuals who were consecutively admitted to the hospital from January 2017 to December 2023 with a diagnosis of AF. A total of 7,456 patients diagnosed with AF were identified, excluding 162 who had a mechanical heart valve or were suffering from moderate-to-severe mitral stenosis, 136 who were admitted with venous thromboembolism, 5,764 who were discharged without receiving rivaroxaban or edoxaban, 118 who had incomplete clinical data (no serum creatinine or weight available), 210 who had multiple admissions (only the first record was retained), and 1,066 patients were ultimately included (Figure 1).

### 2.2 Data collection

Trained personnel collected patient clinical data through the electronic health record. Basic demographic information included department, age, gender, weight, history of bleeding, length of hospital stay, and diagnosis. Laboratory data were collected at the end of hospitalization, including serum creatinine, hemoglobin, and alanine aminotransferase (ALT). Systolic blood pressure and medications were prescribed at discharge. The Cockcroft–Gault formula was used to calculate the creatinine clearance. CHA_2_DS_2_-VA scores were calculated at discharge (1).

### 2.3 Evaluation criteria

The appropriate dosage standards for medications are based on the ESC Atrial Fibrillation Guidelines and the related drug package inserts (Table 1) (1, 15). Underdosing refers to prescribing a medication at a lower dose when the patient dose not fulfill the criteria for dose reduction. Overdosing is characterized by a prescription dose that remains unchanged despite the patient meeting the criteria for dose reduction, or when the total daily dose exceeds the limits recommended in the dosing guidelines.

**Table 1:** Dose selection criteria for NOACs.

Category	Rivaroxaban	Edoxaban
Standard dose	20 mg qd	60 mg qd
Reduced dose	15 mg qd	30 mg qd
Dose-reduction criteria	CrCl 15∼49 mL/min	- creatinine clearance 15–50 mL/min - body weight ≤ 60kg - concomitant use of ciclosporin, dronedarone, erythromycin, or ketoconazole
Contraindicated	CrCl <15 mL/min	CrCl <15 mL/min

### 2.4 Statistical analysis

The Kolmogorov–Smirnov test was utilized to assess the normality of continuous variables. Normally distributed variables were presented as means ± standard deviations, whereas nonnormally distributed variables were expressed as medians and interquartile ranges (IQR). For categorical variables, frequencies and percentages were utilized for description, with either the chi-square test or Fisher's exact test applied for comparisons. To determine the risk factors associated with the inappropriate dose of DOACs, binary logistic regression analysis was performed. Statistically significant variables (*p* < 0.05) in the univariable analysis were subsequently included in the multivariable regression model. All statistical analyses were performed using the Statistical Package for Social Sciences version 22.0, adhering to a significance threshold of *α* = 0.05.

## 3 Results

### 3.1 Study population characteristics

This study included a total of 1,066 patients diagnosed with AF, comprising 852 treated with rivaroxaban and 214 who received edoxaban. The cohort's median age was 69 (IQR, 62–78) years, and 58.7% of them were males. The median length of hospital stay was 9 (IQR, 6–12) days. Catheter ablation was performed in 34.4% of patients. The most common comorbidities were hypertension (71.5%), hyperlipidemia (60.8%), and diabetes (35.0%). The median number of medications prescribed at discharge was 7 (IQR, 5–10). The median CHA_2_DS_2_-VA scores were 3 (IQR, 2–5) (Table 2).

**Table 2:** Factors contributing to inappropriate prescriptions (*N* = 1,066).

Characteristic	Total (*N* = 1,066)	Appropriate dose (*N* = 493)	Inappropriate dose (*N* = 573)	*p*-value
Age, years, median (IQR)	69 (62,78)	67 (60,75)	71 (65,79)	<0.001
Gender, Male, *n* (%)	626 (58.7)	297 (60.2)	329 (57.4)	0.350
Length of stay, days, median (IQR)	9 (6, 12)	8 (6, 11)	9 (6, 13)	0.094
Catheter ablation, *n* (%)	367 (34.4)	205 (41.6)	162 (28.3)	<0.001
Number of medications at discharge, median (IQR)	7 (5,10)	7 (5,9)	8 (5,10)	<0.001
Comorbidities				
Heart failure, *n* (%)	346 (32.5)	152 (30.8)	194 (33.9)	0.293
Hypertension, *n* (%)	762 (71.5)	328 (66.5)	434 (75.7)	<0.001
Diabetes, *n* (%)	373 (35.0)	157 (31.8)	216 (37.7)	0.046
Coronary heart disease, *n* (%)	347 (32.6)	142 (28.8)	205 (35.8)	0.015
Hyperlipidemia, *n* (%)	648 (60.8)	297 (60.2)	351 (61.3)	0.736
Stroke, *n* (%)	234 (22.0)	82 (16.6)	152 (26.5)	<0.001
Hyperuricemia, *n* (%)	250 (23.5)	129 (26.2)	121 (21.1)	0.052
Infectious disease, *n* (%)	240 (22.5)	102 (20.7)	138 (24.1)	0.186
Hypoalbuminemia, *n* (%)	64 (6.0)	27 (5.5)	37 (6.5)	0.502
Anemia, *n* (%)	135 (12.7)	62 (12.6)	73 (12.7)	0.936
COPD, *n* (%)	30 (2.8)	10 (2.0)	20 (3.5)	0.150
Malignancy, *n* (%)	55 (5.2)	25 (5.1)	30 (5.2)	0.904
Bleeding history, *n* (%)	78 (7.3)	25 (5.1)	53 (9.2)	0.009
SBP at discharge, mmHg, median (IQR)	124 (115, 132)	123 (115, 130)	124 (116, 132)	0.05
ALT at discharge^a^, median (IQR)	19 (14, 29)	20 (14, 30)	19 (13, 27)	0.047
Hemoglobin count at discharge^b^, median (IQR)	134 (121, 146)	135 (122, 148)	133 (120, 145)	0.045
CrCl, mL/min, median (IQR)	75.4 (56.6, 94.6)	80.1 (55.3, 102.8)	72.0 (57.6, 88.8)	<0.001
CHA_2_DS_2_-VA, median (IQR)	3 (2, 5)	3 (1, 4)	4 (2, 5)	<0.001
Medications at discharge				
Antiplatelet drugs, *n* (%)	169 (15.9)	57 (11.6)	112 (19.5)	<0.001
Amiodarone, *n* (%)	167 (15.7)	94 (19.1)	73 (12.7)	0.005
Dronedarone, *n* (%)	26 (2.4)	19 (3.9)	7 (1.2)	0.005
Propafenone, *n* (%)	26 (2.4)	12 (2.4)	14 (2.4)	0.992
*β*-blocker, *n* (%)	645 (60.5)	302 (61.3)	343 (59.9)	0.642
Digoxin, *n* (%)	72 (6.8)	34 (6.9)	38 (6.6)	0.864
Diltiazem, *n* (%)	12 (1.1)	5 (1.0)	7 (1.2)	0.749
Statins, *n* (%)	786 (73.7)	348 (70.6)	438 (76.4)	0.030
ACEI/ARB/ARNI, *n* (%)	536 (50.3)	240 (48.7)	296 (51.7)	0.333
NSAID, *n* (%)	7 (0.7)	1 (0.2)	6 (1.0)	0.099
MRA, *n* (%)	171 (16.0)	74 (15.0)	97 (16.9)	0.395
Diuretics, *n* (%)	310 (29.1)	135 (27.4)	175 (30.5)	0.258
Steroids, *n* (%)	12 (1.1)	4 (0.8)	8 (1.4)	0.367
PPI, *n* (%)	558 (52.3)	257 (52.1)	301 (52.5)	0.896
Anti-infection drug, *n* (%)	68 (6.4)	26 (5.3)	42 (7.3)	0.171

Data are shown as mean ± standard deviation, medians (first to third quartiles) or *n* (%).

ACEI, angiotensin-converting enzyme inhibitor; ALT, alanine transaminase; ARB, angiotensin II receptor blocker; ARNI, angiotensin receptor neprilysin inhibitor; COPD, chronic obstructive pulmonary disease; CrCl, creatinine clearance; IQR, interquartile range; MRA, mineralocorticoid receptor antagonist; NSAID, non-steroidal anti-inflammatory drug; PPI, proton pump inhibitor; SBP, systolic blood pressure.

a Full data not available: Total *n* = 1,055, Appropriate dose *n* = 487, Inappropriate dose *n* = 568.

b Full data not available: Total *n* = 1,050, Appropriate dose *n* = 484, Inappropriate dose *n* = 566.

### 3.2 Appropriateness of the prescribed rivaroxaban or edoxaban dose levels

**Figure 2:**
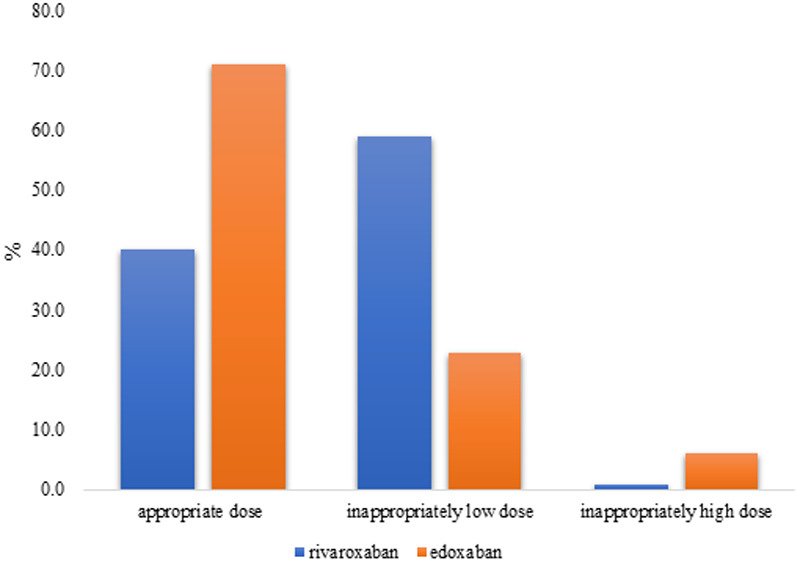
Frequency of appropriate vs. inappropriate dosing.

According to the criteria of the guidelines, 493 patients (46.2%) received the appropriate dose, whereas 573 patients (53.8%) were administered inappropriate doses. Among those prescribed rivaroxaban, 503 (59.0%) were underdosed and eight (0.9%) were overdosed. For those prescribed edoxaban, 49 patients (22.9%) were underdosed and 13 (6.1%) were overdosed. The proportion of appropriate prescriptions was higher for edoxaban (71.0%) than for rivaroxaban (40.0%) (Figure 2).

### 3.3 Factors associated with inappropriate prescriptions

Univariate analysis identified several factors significantly associated with inappropriate prescriptions, including advanced age, a higher total number of medications at discharge, and the presence of comorbidities such as hypertension, diabetes, coronary heart disease, stroke, and bleeding history. In addition, lower ALT, hemoglobin, and creatinine clearance levels, as well as higher CHA_2_DS_2_-VA scores and concomitant prescription of antiplatelet drugs and statins, were associated with inappropriate prescribing. The proportion of patients who underwent catheter ablation, as well as those prescribed amiodarone and dronedarone, was lower in patients prescribed inappropriate medication dosage compared with those who were treated with appropriate regimens (Table 2). Multivariate analysis identified independent factors, including advanced age [adjusted odds ratio (OR) 1.031, 95% confidence interval (CI) 1.010–1.052], the combined use of antiplatelet drugs (adjusted OR 1.649, 95% CI 1.111–2.447), and reduced use of dronedarone (adjusted OR 0.332, 95% CI 0.126–0.877), were associated with inappropriate medication (Table 3).

**Table 3:** Multivariable logistic regression analysis of factors associated with inappropriate of DOACs.

Variable	Adjusted OR (95% CI)	*P*-value
Age	1.031 (1.010–1.052)	0.004
Catheter ablation	0.991 (0.710–1.382)	0.956
Number of medications	0.989 (0.943–1.037)	0.647
Hypertension	1.212 (0.863–1.702)	0.267
Diabetes	0.985 (0.706–1.374)	0.930
Coronary heart disease	0.881 (0.621–1.248)	0.474
Stroke	1.293 (0.836–2.002)	0.248
Bleeding history	1.322 (0.786–2.223)	0.293
ALT	1.000 (0.993–1.006)	0.883
Hemoglobin count	1.003 (0.995–1.010)	0.481
CrCl	1.001 (0.996–1.007)	0.624
CHA2DS2-VA	1.092 (0.920–1.298)	0.314
Antiplatelet drugs	1.649 (1.111–2.447)	0.013
Amiodarone	0.696 (0.471–1.028)	0.069
Dronedarone	0.332 (0.126–0.877)	0.026
Statins	0.942 (0.689–1.289)	0.710

ALT, alanine transaminase; CrCl, creatinine clearance.

Univariate analysis identified the following factors significantly associated with rivaroxaban underdosing: older age, the higher total number of medications at discharge, hypertension, stroke, bleeding history, lower hemoglobin levels, and creatinine clearance, higher CHA_2_DS_2_-VA scores, and concurrent prescription of antiplatelet drugs and nonsteroidal anti-inflammatory drugs (NSAIDs). Among those receiving inappropriate medications, a lower percentage of patients underwent catheter ablation or were prescribed amiodarone compared with those receiving appropriate medication (Table 4). In the multivariate analysis, older age (adjusted OR 1.053, 95% CI 1.029–1.078) and the combined use of antiplatelet drugs (adjusted OR 1.949, 95% CI 1.238–3.069) were independent factors found to be associated with rivaroxaban underdosing (Table 5).

**Table 4:** Factors influencing inadequate low dose of rivaroxaban (*N* = 844).

Characteristic	Total (*N* = 844)	Appropriate dose (*N* = 341)	Inappropriate low dose (*N* = 503)	*p*-value
Age, years, median (IQR)	69 (62, 77)	65 (57, 71)	71 (65, 79)	<0.001
Gender, Male, *n* (%)	512 (60.7)	216 (63.3)	296 (58.8)	0.189
Length of stay, days, median (IQR)	9 (6,12)	9 (6,12)	9 (6,12)	0.499
Catheter ablation, *n* (%)	288 (34.1)	150 (44.0)	138 (27.4)	<0.001
Number of medications at discharge, median (IQR)	7 (5,10)	6 (5,9)	7 (5,10)	<0.001
Comorbidities				
Heart failure, *n* (%)	268 (31.8)	100 (29.3)	168 (33.4)	0.212
Hypertension, *n* (%)	609 (72.2)	231 (67.7)	378 (75.1)	0.018
Diabetes, *n* (%)	291 (34.5)	107 (31.4)	184 (36.6)	0.119
Coronary heart disease, *n* (%)	282 (33.4)	104 (30.5)	178 (35.4)	0.140
Hyperlipidemia, *n* (%)	517 (61.3)	208 (61.0)	309 (61.4)	0.899
Stroke, *n* (%)	186 (22.0)	56 (16.4)	130 (25.8)	0.001
Hyperuricemia, *n* (%)	201 (23.8)	95 (27.9)	106 (21.1)	0.023
Infectious disease, *n* (%)	177 (21.0)	62 (18.2)	115 (22.9)	0.101
Hypoalbuminemia, *n* (%)	46 (5.5)	17 (5.0)	29 (5.8)	0.624
Anemia, *n* (%)	88 (10.4)	30 (8.8)	58 (11.5)	0.202
COPD, *n* (%)	23 (2.7)	5 (1.5)	18 (3.6)	0.064
Malignancy, *n* (%)	48 (5.7)	21 (6.1)	27 (5.4)	0.627
Bleeding history, *n* (%)	61 (7.2)	16 (4.7)	45 (8.9)	0.019
SBP at discharge, mmHg, median (IQR)	124 (116, 132)	123 (116, 130)	125 (116, 133)	0.096
ALT at discharge^a^, median (IQR)	19 (14, 29)	20 (14, 31)	19 (13, 27)	0.067
Hemoglobin count at discharge^b^, median (IQR)	135 (122, 147)	137 (124, 150)	133 (121, 146)	0.006
CrCl, mL/min, median (IQR)	76.64 (58.4, 96.0)	85.77 (60.0, 108.9)	72.28 (58.0, 89.2)	<0.001
CHA_2_DS_2_-VA, median (IQR)	3 (2, 5)	2 (1, 4)	4 (2, 5)	<0.001
Medications at discharge				
Antiplatelet drugs, *n* (%)	139 (16.5)	36 (10.6)	103 (20.5)	<0.001
Amiodarone, *n* (%)	142 (16.8)	73 (21.4)	69 (13.7)	0.003
Dronedarone, *n* (%)	3 (0.4)	1 (0.3)	2 (0.4)	0.803
Propafenone, *n* (%)	17 (2.0)	7 (2.1)	10 (2.0)	0.948
β-blocker, *n* (%)	514 (60.9)	206 (60.4)	308 (61.2)	0.810
Digoxin, *n* (%)	55 (6.5)	22 (6.5)	33 (6.6)	0.950
Diltiazem, *n* (%)	10 (1.2)	3 (0.9)	7 (1.4)	0.500
Statins, *n* (%)	626 (74.2)	242 (71.0)	384 (76.3)	0.080
ACEI/ARB/ARNI, *n* (%)	420 (49.8)	160 (46.9)	260 (51.7)	0.174
NSAID, *n* (%)	6 (0.7)	0 (0.0)	6 (1.2)	0.043
MRA, *n* (%)	138 (16.4)	52 (15.2)	86 (17.1)	0.476
Diuretics, *n* (%)	241 (28.6)	88 (25.8)	153 (30.4)	0.146
Steroids, *n* (%)	8 (0.9)	3 (0.9)	5 (1.0)	0.866
PPI, *n* (%)	440 (52.1)	182 (53.4)	258 (51.3)	0.553
Anti-infection drug, *n* (%)	46 (5.5)	14 (4.1)	32 (6.4)	0.157

Data are shown as mean ± standard deviation, medians (first to third quartiles) or n(%).

ACEI, angiotensin-converting enzyme inhibitor; ALT, alanine transaminase; ARB, angiotensin II receptor blocker; ARNI, angiotensin receptor neprilysin inhibitor; COPD, chronic obstructive pulmonary disease; CrCl, creatinine clearance; IQR, interquartile range; MRA, mineralocorticoid receptor antagonist; NSAID, non-steroidal anti-inflammatory drug; PPI, proton pump inhibitor; SBP, systolic blood pressure.

a Full data not available: Total *n* = 839, Appropriate dose *n* = 339, Inappropriate low dose *n* = 500.

b Full data not available: Total *n* = 831, Appropriate dose *n* = 335, Inappropriate low dose *n* = 496.

**Table 5:** Multivariable logistic regression analysis of factors associated with inappropriate low dose of rivaroxaban.

Variable	Adjusted OR (95% CI)	*P*-value
Age	1.053 (1.029–1.078)	<0.001
Catheter ablation	0.857 (0.587–1.252)	0.429
Number of medications	1.000 (0.946–1.057)	0.996
Hypertension	1.087 (0.745–1.587)	0.665
Stroke	1.392 (0.871–2.226)	0.167
Hyperuricemia	0.718 (0.502–1.026)	0.069
Bleeding history	1.153 (0.615–2.161)	0.657
Hemoglobin count	1.002 (0.994–1.010)	0.631
CrCl	1.001 (0.994–1.008)	0.744
CHA2DS2-VA	1.017 (0.868–1.192)	0.834
Antiplatelet drugs	1.949 (1.238–3.069)	0.004
Amiodarone	0.750 (0.484–1.163)	0.199

CrCl, creatinine clearance; NSAID, non-steroidal anti-inflammatory drug.

The variable “NSAID use” was excluded from the multivariable logistic regression model due to complete/quasi-complete separation in the data, which precluded stable estimation of its effect and led to unreliable odds ratios and confidence intervals.

Factors that were significantly associated with edoxaban underdosing in the univariate analysis included longer hospitalization; higher total number of medications at discharge; hypertension, coronary heart disease, and stroke comorbidities; higher CHA_2_DS_2_-VA scores; and combined use of anti-infection drugs. The proportion of patients who underwent catheter ablation and were prescribed dronedarone or β-blockers was lower among those receiving inappropriate medication compared with those receiving appropriate medication (Table 6). In the multivariate analysis, the presence of hypertension was independently associated with edoxaban underdosing (adjusted OR 3.529, 95% CI 1.304–9.548). β-blocker use was associated with a lower risk of inappropriate dosing (adjusted OR 0.427, 95% CI 0.203–0.897) (Table 7).

**Table 6:** Factors influencing inadequate low dose of edoxaban (*N* = 201).

Characteristic	Total (*N* = 201)	Appropriate dose (*N* = 152)	Inappropriate low dose (*N* = 49)	*p*-value
Age, years, median (IQR)	73 (65.5, 82.0)	72 (64.0, 80.8)	74 (68.5, 82.5)	0.125
Gender, Male, *n* (%)	107 (53.2)	81 (53.3)	26 (53.1)	0.978
Length of stay, days, median (IQR)	8 (6, 11)	8 (6, 10)	10 (7, 14)	0.011
Catheter ablation, *n* (%)	66 (32.8)	55 (36.2)	11 (22.4)	0.075
Number of medications at discharge, median (IQR)	8 (6, 10)	7 (5, 10)	9 (7, 12)	0.001
Comorbidities				
Heart failure, *n* (%)	74 (36.8)	52 (34.2)	22 (44.9)	0.177
Hypertension, *n* (%)	139 (69.2)	97 (63.8)	42 (85.7)	0.004
Diabetes, *n* (%)	73 (36.3)	50 (32.9)	23 (46.9)	0.075
Coronary heart disease, *n* (%)	60 (29.9)	38 (25.0)	22 (44.9)	0.008
Hyperlipidemia, *n* (%)	118 (58.7)	89 (58.6)	29 (59.2)	0.938
Stroke, *n* (%)	43 (21.4)	26 (17.1)	17 (34.7)	0.009
Hyperuricemia, *n* (%)	46 (22.9)	34 (22.4)	12 (24.5)	0.759
Infectious disease, *n* (%)	60 (29.9)	40 (26.3)	20 (40.8)	0.054
Hypoalbuminemia, *n* (%)	17 (8.5)	10 (6.6)	7 (14.3)	0.092
Anemia, *n* (%)	45 (22.4)	32 (21.1)	13 (26.5)	0.424
COPD, *n* (%)	6 (3.0)	5 (3.3)	1 (2.0)	0.655
Malignancy, *n* (%)	7 (3.5)	4 (2.6)	3 (6.1)	0.246
Bleeding history, *n* (%)	14 (7.0)	9 (5.9)	5 (10.2)	0.306
SBP at discharge, mmHg, median (IQR)	123 (114.5, 131.0)	122 (113.0, 130.0)	123 (119.0, 132.0)	0.145
ALT at discharge^a^, median (IQR)	19 (14, 27)	19.5 (15, 27)	19 (12, 26)	0.536
Hemoglobin count at discharge^b^, mean ± standard	129.5 ± 20.3	130.2 ± 19.4	127.2 ± 23.0	0.369
CrCl, mL/min, median (IQR)	72.3 (50.0, 88.4)	71.5 (48.6, 88.4)	74.1 (60.1, 90.0)	0.457
CHA_2_DS_2_-VA, median (IQR)	3 (2, 5)	3 (2, 4)	5 (3, 6)	<0.001
Medications at discharge				
Antiplatelet drugs, *n* (%)	29 (14.4)	21 (13.8)	8 (16.3)	0.664
Amiodarone, *n* (%)	23 (11.4)	21 (13.8)	2 (4.1)	0.063
Dronedarone, *n* (%)	18 (9.0)	18 (11.8)	0 (0.0)	0.012
Propafenone, *n* (%)	7 (3.5)	5 (3.3)	2 (4.1)	0.793
β-blocker, *n* (%)	118 (58.7)	96 (63.2)	22 (44.9)	0.024
Digoxin, *n* (%)	17 (8.5)	12 (7.9)	5 (10.2)	0.613
Diltiazem, *n* (%)	2 (1.0)	2 (1.3)	0 (0.0)	>0.999
Statins, *n* (%)	147 (73.1)	106 (69.7)	41 (83.7)	0.056
ACEI/ARB/ARNI, *n* (%)	106 (52.7)	80 (52.6)	26 (53.1)	0.958
NSAID, *n* (%)	1 (0.5)	1 (0.7)	0 (0.0)	>0.999
MRA, *n* (%)	31 (15.4)	22 (14.5)	9 (18.4)	0.512
Diuretics, *n* (%)	66 (32.8)	47 (30.9)	19 (38.8)	0.309
Steroids, *n* (%)	3 (1.5)	1 (0.7)	2 (4.1)	0.148
PPI, *n* (%)	104 (51.7)	75 (49.3)	29 (59.2)	0.231
Anti-infection drug, *n* (%)	22 (10.9)	12 (7.9)	10 (20.4)	0.015

Data are shown as mean ± standard deviation, medians (first to third quartiles) or *n* (%).

ACEI, angiotensin-converting enzyme inhibitor; ALT, alanine transaminase; ARB, angiotensin II receptor blocker; ARNI, angiotensin receptor neprilysin inhibitor; COPD, chronic obstructive pulmonary disease; CrCl, creatinine clearance; IQR, interquartile range; MRA, mineralocorticoid receptor antagonist; NSAID, non-steroidal anti-inflammatory drug; PPI, proton pump inhibitor; SBP, systolic blood pressure.

a Full data not available: Total *n* = 195, Appropriate dose *n* = 148, Inappropriate low dose *n* = 47.

b Full data not available: Total *n* = 198, Appropriate dose *n* = 149, Inappropriate low dose *n* = 49.

**Table 7:** Multivariable logistic regression analysis of factors associated with inappropriate low dose of edoxaban.

Variable	Adjusted OR (95% CI)	*P*-value
Length of stay	1.045 (0.961–1.135)	0.304
Number of medications	1.050 (0.931–1.184)	0.426
Hypertension	3.529 (1.304–9.548)	0.013
Coronary heart disease	1.783 (0.690–4.605)	0.232
Stroke	1.963 (0.665–5.797)	0.222
CHA2DS2-VA	0.924 (0.647–1.319)	0.663
Dronedarone	0.000 (0.000-)	0.998
β-blocker	0.427 (0.203–0.897)	0.025
Anti-infection drug	2.194 (0.754–6.379)	0.149

## 4 Discussion

Our findings provide real-world evidence that may facilitate optimal treatment choice. The key results of this study are summarized as follows: (1) About 53.8% of patients with AF were given inappropriate dosing, with 51.8% treated with underdoses and 2.0% with overdoses. Among those prescribed rivaroxaban, 59.0% were underdosed, and 0.9% were overdosed. Of the patients prescribed edoxaban, 22.9% were underdosed and 6.1% were overdosed. (2) Several independent factors were found to be associated with inappropriate medication dosing, including advanced age, the concurrent use of antiplatelet drugs, and reduced usage of dronedarone. (3) Older age and the concurrent usage of antiplatelet agents were factors independently associated with rivaroxaban underdosing. For edoxaban underdosing, the presence of hypertension was identified as a significant factor. Additionally, β-blocker administration was found to correlate with a lower risk of underdosing.

Rivaroxaban received its first clinical approved in China in 2009, while edoxaban was approved for clinical use in 2018. This discrepancy might explain the lower prescription rates of edoxaban observed in our study. A cross-sectional study conducted in Belgium indicated that inappropriate prescribing occurred in 16.9% of patients, with underdosing (9.7%) being more prevalent than overdosing (6.9%) (16). An Australia study revealed that 28.9% of patients with AF were prescribed inappropriate doses. Among them, underdosing was the predominant issue, with 22.9% for apixaban, 7.1% for dabigatran, and 25.1% for rivaroxaban (17). Similarly, the ANATOLIA-AF study reported that 24.9% of participants did not receive the appropriate DOAC dosage (18). A French registration study also found that 46% of patients were treated with inappropriate underdosing, with a higher prevalence observed among those reveiving apixaban (19). A retrospective registry revealed that 57.3% of patients were administered off-label doses of DOACs, including rivaroxaban and dabigatran (20). A study utilizing medical data from a multicenter health care system in Taiwan revealed that approximately 27% and 5% of AF patients were treated with underdosing and overdosing of NOACs, respectively (21). The variation in the prevalence of inappropriate DOAC dosing observed in real-world studies may be attributed to several factors, including the types of NOACs assessed, differences in the criteria of appropriate doses, ethnic differences in the bleeding risk associated with DOACs, availability of targeted anticoagulation reversal agents, and level of physicians' knowledge (22, 23).

Previous studies have indicated that patients receiving off-label DOAC doses do not receive the full benefit of anticoagulation (24). The GARFIELD-AF (Global Anticoagulant Registry in the FIELD-AF) study, conducted between 2013 and 2016, revealed that 72.9% received the recommended dosing, whereas 23.2% were underdosed and 3.8% were overdosed. Non-recommended dosing was associated with a higher risk of all-cause mortality when compared with the recommended dosing (14). A retrospective registry study found that DOACs administered at off-label underdosed were linked to a notably lower risk of major bleeding and all-cause mortality compared with on-label doses. However, no significant differences were observed in the risks of thrombotic events or minor bleeding (20). A systematic review assessed the clinical efficacy and safety of low-dose DOAC treatment vs. on-label dosing in Asian patients with AF. The outcomes of the low-dose treatment did not display significant differences when compared to the standard-dose treatment (25). A study conducted in a tertiary medical center in Taiwan revealed that off-label low-dosing of rivaroxaban should be avoided due to the higher risk of ischemic stroke, without a corresponding reduction in the risk of intracranial hemorrhage when compared to on-label dosing (26).

The reported clinical factors associated with inappropriate dosing vary between studies. Our results indicate that advanced age and the concurrent use of antiplatelet drugs are independent factors influencing inappropriate medication dosing and the underdosing of rivaroxaban.

Previous studies have established that advanced age is an independent risk factor for inappropriate medication use (18, 19, 27). Our present study observed results consistent with these findings because of the increased occurrence of bleeding events in elderly patients, who frequently present with multiple comorbidities, polypharmacy, low weight, frailty, and falls. Our study found that older age was also an independent risk factor associated with rivaroxaban underdosing; however, it was not recognized as a risk factor for edoxaban underdosing. In addition, the incidence of edoxaban underdosing in this study was lower than that of rivaroxaban, which may be associated with edoxaban showing a significantly lower risk of bleeding (28, 29). Another study indicated that the net clinical benefit is more pronounced in the elderly population receiving standard DOAC doses compared to those treated with lower doses (30).

Previous studies have demonstrated that the risk of bleeding increases with the concomitant use of DOACs and antiplatelet agents, and they are well-known in clinical practice (31). Concerns regarding the risk of bleeding may lead clinicians to prescribe low-dose DOACs. Previous guidelines recommended that clinicians utilize the HAS-BLED scores to assess the risk of bleeding in patients with AF. The HAS-BLED score awards 1 point for the use of an antiplatelet drug (15). However, the use of bleeding risk scores was not recommended by the new guidelines; instead, they suggest that modifiable bleeding risk factors should be managed to improve safety (1).

This study revealed that patients using amiodarone and dronedarone were more likely to be prescribed standard DOAC doses, a finding that may differ from previous studies (18). Amiodarone and dronedarone are known potential inhibitors of P-glycoprotein (P-gp) and CYP3A4, which might be associated with an increased risk of bleeding when used with DOACs (32). The results may be attributed to closer monitoring or stricter medication management during hospitalization for patients who underwent catheter ablation. It is important to note that NOACs do not require frequent INR monitoring which benefits patients, but also imposes a risk of clinical consequences due to unrecognized interactions.

Hypertension is a risk factor for inappropriate medication dosing and rivaroxaban underdosing. It has also been identified as an independent risk factor for edoxaban underdosing. Among patients diagnosed with AF, hypertension has been associated with an increased risk of stroke, heart failure, and major bleeding (33, 34). In this population, hypertension frequently exists with other risk factors, such as coronary heart disease and stroke. Additionally, factors such as lower hemoglobin, reduced creatinine clearance, a bleeding history, and a higher CHA_2_DS_2_-VASc score are also important risk factors for inappropriate medication dosing, as reported in previous studies (19, 35, 36). In our study, these factors were identified as univariate variables associated with inappropriate medication dosing; however, the multivariate analysis did not reveal any statistically significant differences.

The unexpected observation regarding edoxaban was that the use of β-blockers was associated with appropriate prescriptions. We hypothesize that this finding may be attributed to the absence of drug interactions with edoxaban, which alleviates concerns about bleeding risks. Conversely, previous studies have reported NSAIDs as factors that could increase the risks associated with low-dose NOAC use due to their potential to increase bleeding risk (18, 37). However, this study reported that NSAIDs exhibited a significant difference in the univariate analysis, but no statistical difference was observed in the multivariate analysis, which may be due to the small number of patients using NSAIDs.

Despite their numerous advantages, NOACs present significant safety challenges, particularly regarding potential drug interactions in patients undergoing polypharmacy and the absence of specific monitoring indicators. While NOACs have fewer drug-drug interactions than warfarin—a clear benefit—this advantage may paradoxically lead clinicians to underestimate the importance of screening for NOAC interactions. Furthermore, since routine therapeutic monitoring is not required for dose adjustment with NOACs, deviations from recommended prescribing information make it difficult to assess the level of anticoagulation. To mitigate inappropriate prescribing, several strategies can be implemented: (1) enhancing pharmacist-led medication reviews to optimize anticoagulant dosing; (2) establishing a multidisciplinary “anticoagulation team” focused on defining optimal management strategies and reducing potentially inappropriate medications, particularly in polypharmacy patients; and (3) implementing computerized decision-support systems (CDSS) to manage anticoagulation and minimize inappropriate anticoagulant prescribing.

This study is, to our knowledge, the first to explore factors associated with inappropriate DOAC dosing in AF patients in China. However, several limitations should be acknowledged. First, this retrospective, single-center study was conducted at a tertiary hospital in Beijing, a major metropolitan area, which may limit generalizability to other regions, countries, or hospital levels and introduces potential selection bias. Second, dabigatran was excluded due to limited availability of only a single dose formulation at our institution, while apixaban was excluded because it was not available at all, both of which constrain the comprehensiveness of our findings. Third, the use of inpatient rather than outpatient data—chosen for superior feature coverage—may limit extrapolation to outpatient settings and real-world practice. Finally, we did not examine the relationship between clinical outcomes and inappropriate DOAC dosing, which warrants investigation in future research.

## 5 Conclusions

This study reveals a high incidence of inappropriate dosing of DOACs in patients with AF in China, with underdosing occurring more frequently, particularly among those prescribed rivaroxaban. Advanced age, concurrent use of antiplatelet medications, and the nonuse of dronedarone were identified as independent factors associated with inappropriate dosing. Currently, patient undertreatment and non-adherence to guidelines remain significant issues, and thereby, the standardization of medication use should be improved.

## Data Availability

The original contributions presented in the study are included in the article/Supplementary Material, further inquiries can be directed to the corresponding author.
